# Critical thinking about health and treatments in the United States: a cross-sectional assessment of parents and undergraduate college students

**DOI:** 10.1186/s12889-025-21291-9

**Published:** 2025-01-27

**Authors:** Vanesa A. Mora Ringle, Astrid Dahlgren, Sarah Rosenbaum, Amanda Jensen-Doss

**Affiliations:** 1 Lehigh University, Bethlehem, USA; 2 OsloMet – Oslo Metropolitan University, Oslo, Norway; 3 Norwegian Institute of Public Health, Oslo, Norway; 4 University of Miami, Coral Gables, USA

**Keywords:** Evidence-based healthcare, Critical health literacy, Critical thinking, Health education, Health claims, Informed health choices

## Abstract

**Background:**

There is an urgent need to study and increase the public’s ability to think critically about health and treatments. Unfortunately, we do not currently have a clear, in-depth understanding of critical thinking about health in the United States, especially its rates among parents and college students, two particularly important groups. This study assessed and characterized critical thinking about health with U. S. parents and college students. We also explored whether critical thinking about health varied as a function of sociodemographic determinants.

**Methods:**

Parents (*N* = 142) and college students (*N* = 145) in the U. S. completed an online test of critical thinking about health, and answered questions about their background.

**Results:**

Both parents and college students in the U. S. struggled to think critically about health and treatments based on various science literacy and evidence-based practice principles. Parents with higher educational attainment had lower critical thinking about health, and college students who reported a liberal political affiliation had higher critical thinking scores.

**Conclusions:**

This investigation demonstrates a need to increase critical thinking about health among U. S. parents and college students so they can be empowered to make informed health choices.

There is an urgent need to study and increase the public’s ability to think critically about health information. More than ever before, people making health decisions are faced with an overabundance of both accurate and inaccurate health information readily available via mass media and the internet [1–3]. Moreover, studies reveal that the public often relies on and trusts anecdotal information more than research evidence [4, 5]. The COVID-19 global pandemic brought these longstanding issues to the forefront, particularly around the public’s COVID-19-related health misconceptions, especially low acceptance of the COVID-19 vaccine [6, 7]. This has precarious public health implications because believing unreliable health claims may lead people to delay pursuing effective services, and/or to pursuing health services that are ineffective or potentially harmful, thus increasing risks and wasting valuable resources. Given the public resources and money spent investigating health issues, the public—regardless of education level and other social determinants—deserves to know and understand the full extent of their health options, so they are empowered to make informed healthcare decisions. Thus, we have an ethical imperative to investigate and disseminate strategies to increase the public’s ability to critically evaluate health information and make informed health choices.

Unfortunately, we do not currently have a clear, in-depth understanding of critical thinking about health and its levels in the United States public. Particularly, it is key to understand the health-related critical thinking of parents and college students. Parents are responsible for healthcare decisions for both themselves and their children, and will at one point or another be confronted with healthcare claims. Young adult college students enrolled in undergraduate programs are newly responsible for making healthcare decisions for themselves and are part of a generation that is distinctly habituated to accessing information through the internet. Furthermore, college students, especially freshmen, are susceptible to stressful circumstances due to their transitional life period, which may impact their health and healthcare-seeking [8]. Thus, making them an important population to study in relation to health literacy. Importantly, critical thinking about health could serve to mitigate health inequities by reducing the detrimental heath impact of other well-known, less malleable social and systemic determinants of health inequities (e.g., socioeconomic status, race/ethnicity, access to education) [9, 10]. Additionally, the recent federal mandate to make federally-funded research publications freely available and easily accessible by the public [11] means that people will likely be making more use of their health-related critical thinking abilities. Overall, critical thinking about health may empower the public to have greater control over their health by facilitating informed health decision-making.

Critical thinking about health is largely understood in the literature as critical health literacy, which is the ability to access, understand, and critically evaluate various sources of health information, and engage in shared-decision making [12, 13]. Critical thinking about health is also interrelated with science literacy, as it involves grasping and applying evidence-based practice principles [13]. The current paper focuses on a conceptualization of critical thinking about health that includes critically evaluating claims about treatment effectiveness and the effects of treatments based on a set of evidence-based practice principles known as the IHC Key Concepts. The use of Key Concepts is supported by the Deweyan premise that we need points of reference (or concepts) from which to engage in critical thinking, especially when we need to do so repeatedly in new situations [13]. Henceforth we refer to these combined abilities as critical thinking about health.

Critical thinking about health resources for the public are scarce. Multidisciplinary researchers from the Informed Health Choices Project (IHC, https://www.informedhealthchoices.org) are pioneering efforts to address this gap by creating, testing, and validating open access critical thinking resources for various members of the public including children, parents, and other adults [14–20]. IHC researchers first developed a framework of principles—the IHC Key Concepts—which can be used as a point of reference when engaging in critical thinking about health claims, including deciding what to believe and act upon [13, 21, 22]. At the time of this study, there were 34 IHC Key Concepts (outlined in Table 1), divided into 3 categories: (1) *Recognizing an unreliable basis for a claim, (2) Understanding whether treatment comparisons are fair and reliable, and (3) Making informed choices* [22]. The IHC Key Concepts can be used to design school curricula, other learning resources, and evaluation instruments, such as the Claim Evaluation Tools. The Claim Evaluation Tools is an ever-growing item bank of over 100 decision-based, multiple-choice questions designed to measure people’s understanding and ability to use the IHC Key Concepts [23, 24]. This IHC tool has been used to assess the skills of specific populations as well as to measure the effects of interventions to improve critical thinking about health [14, 15, 17, 19].

**Table 1:** Informed Health choices Key concepts (Austvoll-Dahlgren et al., 2015)

**Recognizing an unreliable basis for a claim**
1.1 Treatments may be harmful
1.2 Personal experiences or anecdotes (stories) are an unreliable basis for assessing the effects of most treatments
1.3 An ‘outcome’ may be associated with a treatment, but not caused by the treatment
1.4 Widely used treatments or treatments that have been used for a long time are not necessarily beneficial or safe
1.5 New, brand-named, or more expensive treatments may not be better than available alternatives
1.6 Opinions of experts or authorities do not alone provide a reliable basis for deciding on the benefits and harms of treatments
1.7 Conflicting interests may result in misleading claims about the effects of treatments
1.8 Increasing the amount of a treatment does not necessarily increase the benefits of a treatment and may cause harm
1.9 Earlier detection of disease is not necessarily better
1.10 Hope or fear can lead to unrealistic expectations about the effects of treatments
1.11 Beliefs about how treatments work are not reliable predictors of the actual effects of treatments
1.12 Large, dramatic effects of treatments are rare
**Understanding whether comparisons are fair and reliable**
2.1 Evaluating the effects of treatments requires appropriate comparisons
2.2 Apart from the treatments being compared, the comparison groups need to be similar (i.e. ‘like needs to be compared with like’)
2.3 People’s outcomes should be counted in the group to which they were allocated
2.4 People in the groups being compared need to be cared for similarly (apart from the treatments being compared)
2.5 If possible, people should not know which of the treatments being compared they are receiving
2.6 Outcomes should be measured in the same way (fairly) in the treatment groups being compared
2.7 It is important to measure outcomes in everyone who was included in the treatment comparison groups
2.8 The results of single comparisons of treatments can be misleading
2.9 Reviews of treatment comparisons that do not use systematic methods can be misleading
2.10 Unpublished results of fair comparisons may result in biased estimates of treatment effects
2.11 Results for a selected group of people within a systematic review of fair comparisons of treatments can be misleading
2.12 Relative effects of treatments alone can be misleading
2.13 Average differences between treatments can be misleading
2.14 Small studies in which few outcome events occur are usually not informative and the results may be misleading
2.15 The use of p-values to indicate the probability of something having occurred by chance may be misleading; confidence intervals are more informative
2.16 Saying that a difference is statistically significant or that it is not statistically significant can be misleading
2.17 A lack of evidence is not the same as evidence of “no difference”
**Making informed choices**
3.1 A systematic review of fair comparisons of treatments should measure outcomes that are important
3.2 A systematic review of fair comparisons of treatments in animals or highly selected groups of people may not be relevant
3.3 The treatments evaluated in fair comparisons may not be relevant or applicable
3.4 Well done systematic reviews often reveal a lack of relevant evidence, but they provide the best basis for making judgements about the certainty of the evidence
3.5 Decisions about treatments should not be based on considering only their benefits

No study to date has examined critical thinking about health in the U. S. public using the Claim Evaluation Tools item bank. One cross-sectional study investigated critical thinking about health at the population level with Norwegian adults [18].This study assessed Norwegians’ ability to think critically about health based on 30 IHC Key Concepts, and found that the adjusted proportion of Norwegian adults who provided correct answers exceeded 50% for 17 out of 30 Key Concepts, while fewer than half answered correctly for the remaining 13 concepts. Furthermore, they struggled most with the Key Concept, “Results for a selected group of people within a study can be misleading;” while their critical thinking abilities were best based on the Key Concept, “Increasing the amount of a treatment does not necessarily increase its benefits and may cause harm.”

Examination of sociodemographic characteristics may help clarify variance in critical thinking about health. Particularly, Dahlgren et al. found that higher educational attainment was associated with better critical thinking in their sample of Norwegian adults [18]. In addition, previous studies in related areas, such as science literacy and attitudes, have established a relationship between political ideology and attitudes toward science [25–27]. Notably, in a recent, comprehensive evaluation of the relationships between political ideology, science knowledge, cognitive sophistication, and science beliefs, Pennycook et al. (2022) found basic science knowledge and critical thinking, and not political ideology, to be the most consistent predictors of science beliefs across a variety of topics [27]. Nevertheless, no study to date has examined if and how critical thinking about health varies as a function of political affiliation, nor examined the role of educational attainment in critical thinking about health in the U. S.

## Current study

Critical thinking about health, including the ability to evaluate claims about the effectiveness and effects of treatments, empowers the public to make informed health choices. Given the public health implications of critical thinking about health, and the lack of such studies among U. S. parents and college students, we sought to assess and characterize critical thinking about health with these populations. This study also served as a needs-assessment to inform the development of a critical thinking intervention for the U. S. public. We also aimed to explore whether critical thinking about health varied as a function of sociodemographic factors, including educational attainment, age, income, and political partisanship. Although these analyses were largely exploratory, based on past findings [e.g., 28] indicating that disadvantaged socioeconomic conditions are often related to lower health literacy, we hypothesized that lower educational attainment and lower income would be associated with lower critical thinking about health. We did not have specific directional predictions regarding political ideology given the nuance of past mixed findings (e.g [27, 29, 30]).

## Method

### Participants

We obtained approval from the University of Miami Institutional Review Board prior to beginning study. In total, we recruited 370 adult participants (179 parents; 191 college students) from the United States. Parents participated in the study from August to September 2018 through Amazon’s Mechanical Turk (MTurk), an online labor market where anonymous “workers” receive monetary compensation for completing various research tasks as part of survey, experimental, and intervention studies [31, 32]. College students were recruited through an undergraduate study pool and participated from February to April 2019.

We chose to use MTurk (via CloudResearch - cloudresearch.com) due to MTurk’s demonstrated reliability for expediently and cost-effectively recruiting diverse parents, especially in comparison to other common online recruitment methods such as Listservs [33–35]. CloudResearch’s online platform links to Mturk and provides additional features that facilitate data collection [36]. To participate, parents had to be U. S. residents, have an MTurk approval rating of 98% or higher, and have at least one child below age 18 (as determined by self-report). An Mturk approval score refers to the percentage of tasks that an Mturk participant has successfully completed and that have been approved by researchers. This metric reflects the reliability and quality of a participant’s submissions based on their track record. Higher approval scores are used to ensure data quality by selecting participants with a consistent history of meeting task requirements. Parents received a $2.00 compensation through MTurk for completing study measures [according to MTurk best practice recommendations; see [37]. College student participants were informed of this study through the study participation pool available to undergraduates completing a psychology course at the University [masked for review]. To participate, college students had to be at least 18 years old. They received partial fulfillment of an undergraduate research requirement as compensation.

Out of an abundance of caution and to optimize data quality, we checked study completion length relative to the projected completion time of 20 min, and included an attention check question (Chandler et al. 2019). Accordingly, prior to data analyses, we excluded participants who completed the measures in less than 10 min (half of the projected completion time), and those who failed attention checks. This resulted in samples sizes of 142 parents (79% of total N) and 145 college students (76% of total N).

## Measures

### Demographics and other characteristics

We asked participants to provide information about their age, gender, race/ethnicity, annual household income, and educational attainment. Educational attainment was measured as highest level of education (high school, some college, bachelor’s degree, etc.) for parents, while college students reported on their class standing, since they were still in college. Other variables also differed by sample: we asked only parents about number of children and employment status, and only college students reported their political affiliation, and whether they had children (yes/no).

### Critical thinking about health

**Fig. 1:**
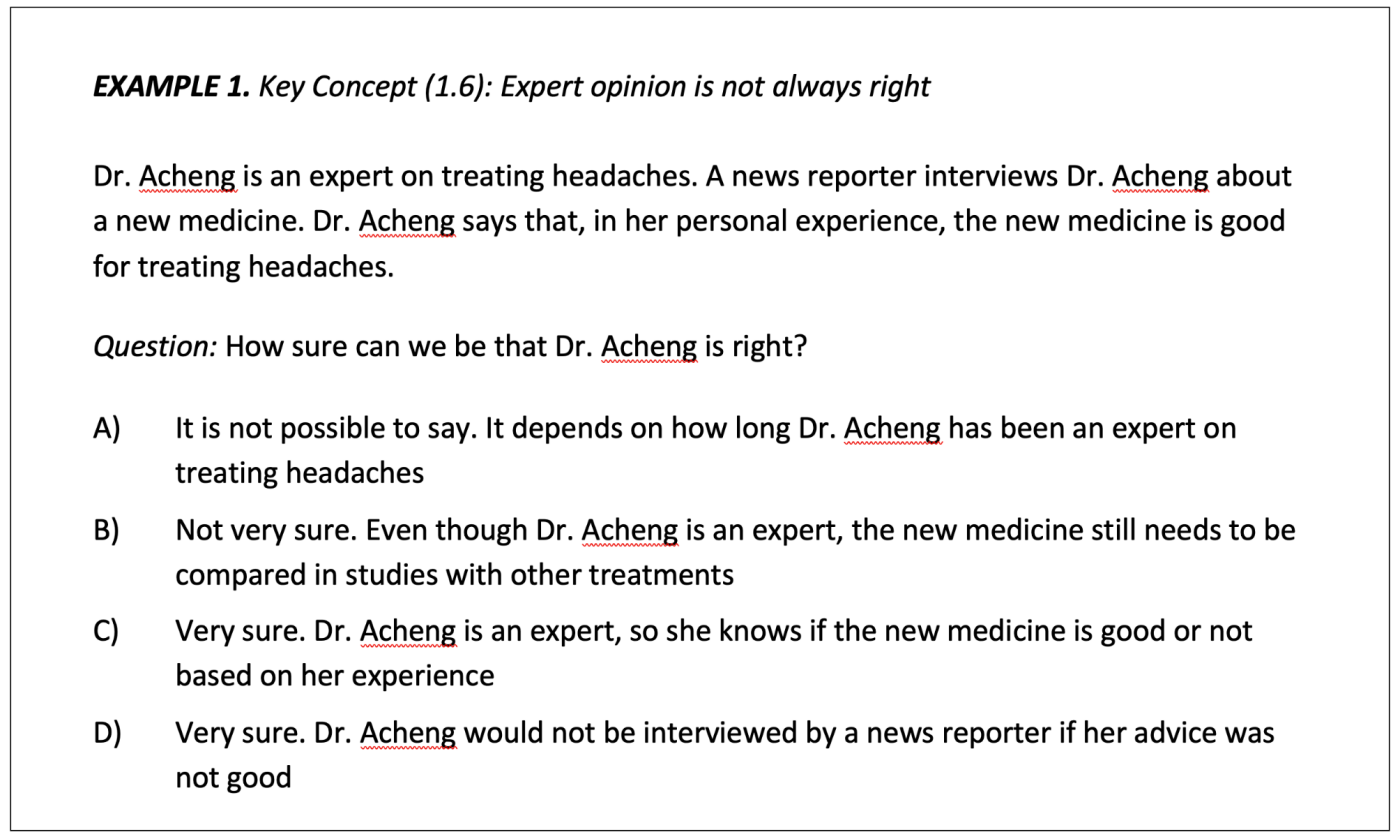
Sample critical thinking questions

We assessed critical thinking related to 26 IHC Key Concepts using 52 multiple choice questions (2 questions per concept) from the Claim Evaluation Tools [23, 24]. We include a sample question in Fig. 1 example 1. This measure is an ever-growing bank of over 100 multiple choice questions that can be used with individuals ages 10 and older, and includes items about effectiveness and the ability to assess claims about treatment effects based on different Key Concepts (see Table 1). Items used in the present study were selected based on their cultural relevance to a U. S. audience, and on how well they performed in a previous validation study [24], in consultation with the measure developer. Items found to be very easy in other similar, high-income countries were not selected. Test organization was such that questions addressing the same Key Concept were not adjacent to each other. Instruments created using questions from the Claim Evaluation Tools item bank have been validated in several contexts and found to have good psychometric properties. Using Cronbach’s alpha, the internal consistency of the scale was excellent (α = 0.93). We also further tested the psychometric properties of this study’s instrument, in the first such evaluation conducted in the U. S.; findings from this evaluation are reported on a separate manuscript.

## Analytic plan

We used SPSS Version 28 to run descriptive statistics and frequencies of demographic variables and proportions of correct responses. We calculated total scores using the 52 original items from the Claim Evaluation Tools. To determine critical thinking per Key Concept, we averaged item-level proportions of correct responses. Additionally, to optimize description and communication of findings, we calculated overall Key Concept averages by aggregating scores across the three Key Concept categories: (1) Recognizing an unreliable basis for a claim, (2) Understanding whether comparisons are fair and reliable, and (3) Making informed health choices. We used T-tests and Pearson correlation coefficients to examine critical thinking differences based on sociodemographic characteristics. Analyses were run separately for parents and college students. Age and household income were entered as continuous variables. Parent educational attainment was entered as a dichotomous variable (bachelor’s degree or more versus less than a bachelor’s degree). Political affiliation was only available for college students and was entered as a categorical variable (e.g., conservative versus not; liberal versus not).

## Results

Parents had a mean age of 37 years, and were 45% female, predominantly White (83%), and 55% had a Bachelor’s or higher degree. College students had a mean age of 19 years, were 56% female, 67% White, 63% were college freshman, and 62% reported a household income of $100,000 or higher. See Table 2 for detailed demographic information.

**Table 2:** Demographic information

	Parents (*N* = 142)	College students (*N* = 145)
Characteristic	Mean (SD, range) or % (n)	
Age	36.86 (7.93, 23–64)	19.21(1.211, 18–23)
Gender		
Female	45.1% (64)	55.9% (81)
Race/Ethnicity*		
African American	7.7% (11)	8.3% (12)
American Indian or Alaska Native	0.7% (1)	1.4 (2)
Asian	5.6% (8)	15.2 (22)
Hispanic	4.2% (6)	24.8% (36)
White	83.1% (118)	66.9% (97)
Highest Level of Education Achieved+		
Some high school, no diploma	0	--
High school	10.6% (15)	--
Some college, no degree	19.7% (28)	--
Associate’s or technical degree	14.8% (21)	--
Bachelor’s degree	43.0% (61)	--
Master’s degree	12.0% (17)	--
Doctoral or other graduate degree	0	--
Class Standing+		
Freshman	--	62.8% (91)
Sophomore	--	23.4% (34)
Junior	--	6.2% (9)
Senior	--	7.6% (11)
Employment Status+		
Currently working	82.4% (117)	--
Unemployed	4.2% (6)	--
Retired	0	--
Homemaker	12.7% (18)	--
Student or other	0.7% (1)	--
Annual Household Income+		
Less than $19,999	3.5% (5)	5.6% (8)
$20,000 to $39,999	27.5% (39)	5.5% (8)
$40,000 to $59,999	25.4% (36)	7.6% (11)
$60,000 to $79,999	18.4% (26)	8.9% (13)
$80,000 to $99,999	9.9% (14)	8.9% (13)
More than $100,000	15.5% (22)	62.1% (90)
Political Affiliation+		
Liberal	--	37.9% (55)
Moderate	--	43.4% (63)
Conservative	--	13.1% (19)
Other	--	4.8% (7)
Children #+	2 (1.31, 1–10)	--

*Indicates percentages do not sum to 100 due to overlap across categories

+ Data missing (*n* = 1–2)

### Parents’ critical thinking about health

**Fig. 2:**
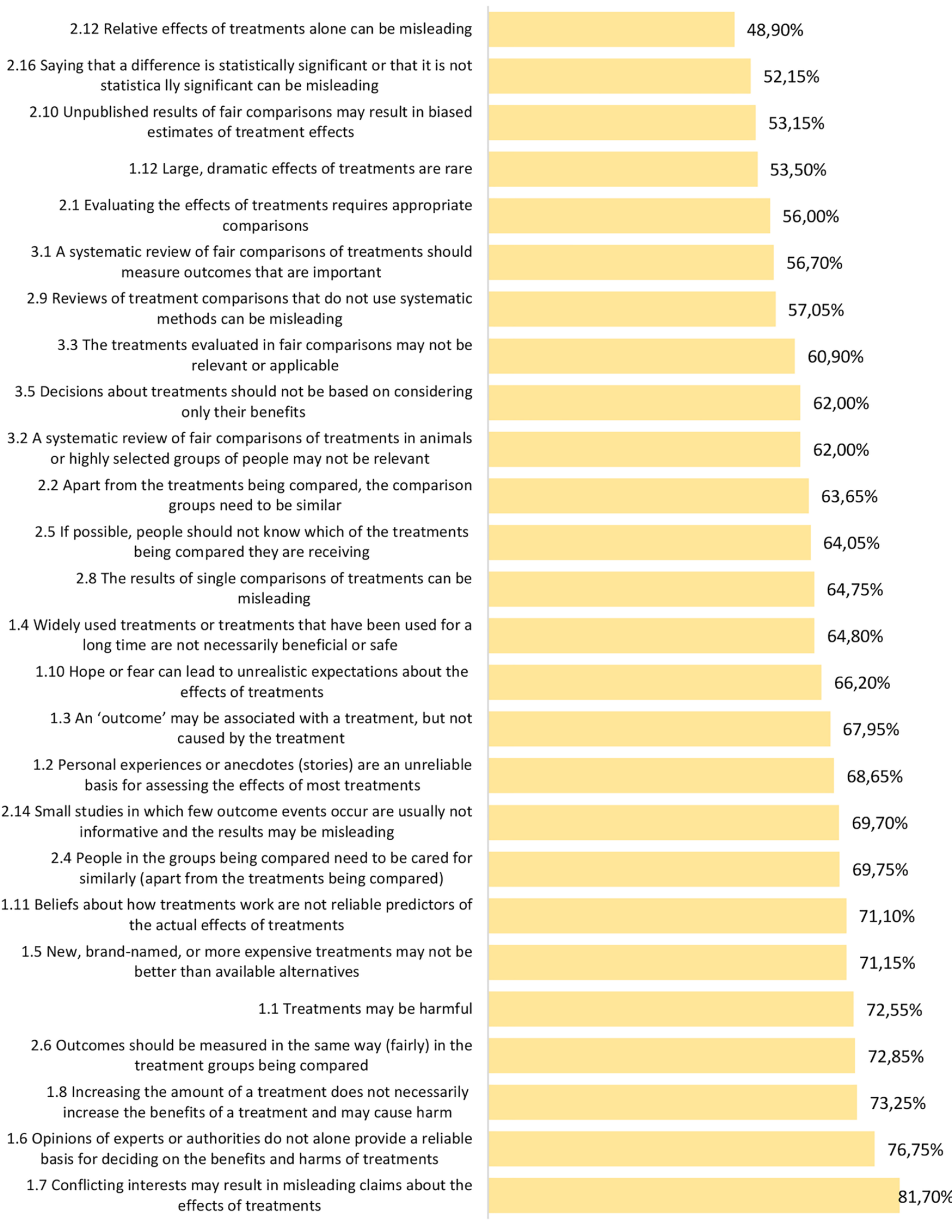
Average of proportions of parents’ correct responses in two questions per Key Concept

We found that, despite being a relatively educated sample (55% with a bachelor’s degree or higher), parents struggled to engage in critical thinking about health with an average proportion of correct responses of 64.67% (Mean = 33.63 out of 52 questions; SD = 11.4; Range = 10–51). Parents’ averaged scores across the three Key Concept categories were as following from highest to lowest: Recognizing an unreliable basis for a claim (0.69), Understanding whether comparisons are fair and reliable (0.61), and Making informed health choices (0.60). Out of the 26 Key Concepts covered, the Key Concept parents understood and applied best was, “Conflicting interests may result in misleading claims about the effects of treatments” (81.70% correct responses). Parents most struggled to think critically based on the Key Concept: “Relative effects of treatments alone can be misleading” (48.90% correct responses). See Fig. 2 for parent results across each Key Concept.

### College students’ critical thinking about health

**Fig. 3:**
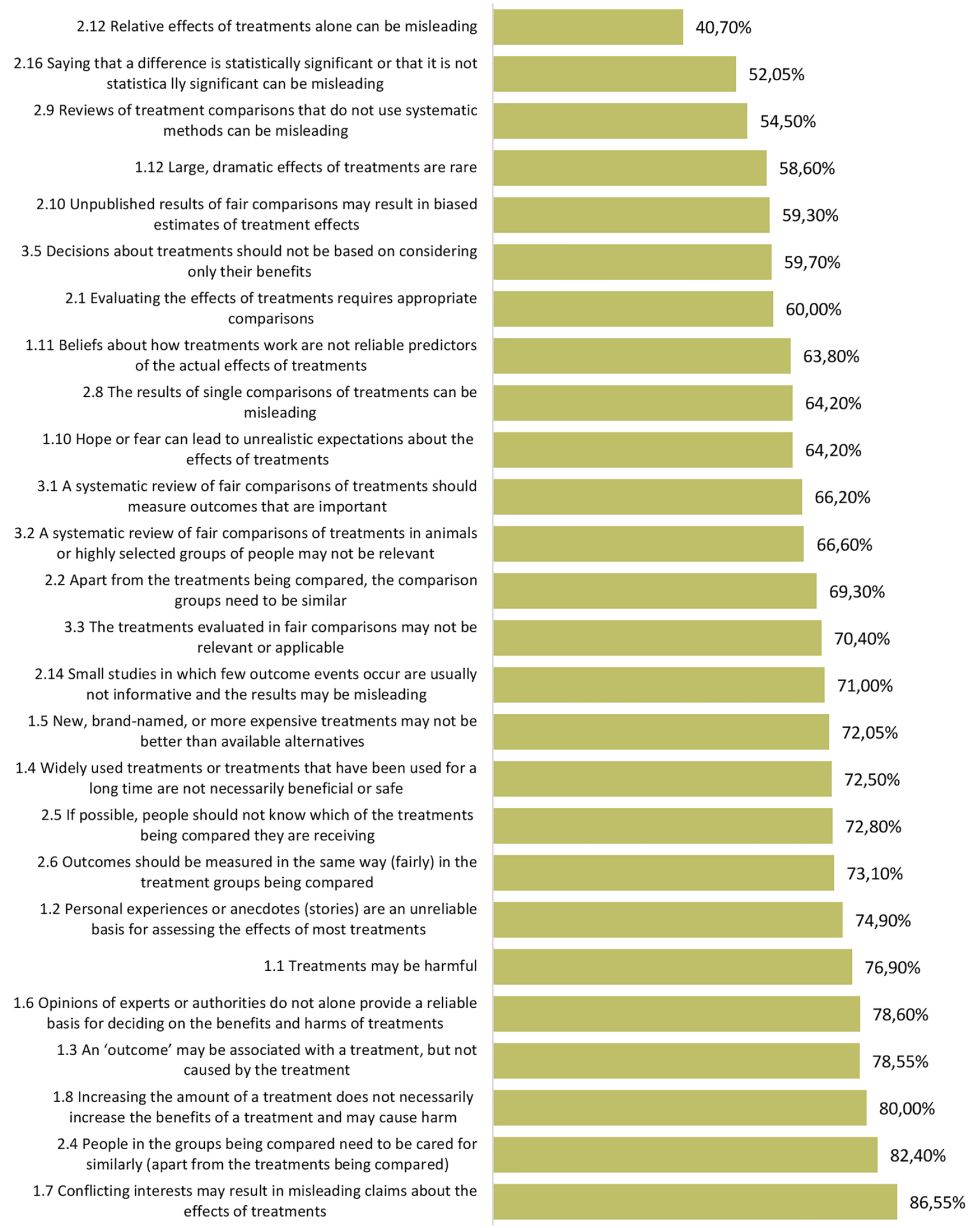
Average of proportions of college students’ correct responses in two questions per Key Concept

College students’ critical thinking score was 68% (Mean = 35.37 out of 52 questions; SD = 8.0; Range = 15–50). Although higher than the parents’ overall score, this difference did not reach statistical significance. College students’ averaged scores across the three Key Concept categories were as following from highest to lowest: Recognizing an unreliable basis for a claim (0.72), Making informed health choices (0.66), Understanding whether comparisons are fair and reliable (0.64). The Key Concept college students critically applied best was “Conflicting interests may result in misleading claims about the effects of treatments” (86.60% correct responses). Like parents, college students struggled most with “Relative effects of treatments alone can be misleading” (40.70% correct responses). See Fig. 3 for college student results across each Key Concept.

### Exploratory analyses: Sociodemographic factors and critical thinking about health

Parents with a bachelor’s degree (critical thinking mean score = 30.7) had significantly lower critical thinking scores than parents with lower educational attainment, whose critical thinking mean score was 12% points higher (critical thinking mean score = 37.2) (t[140] = 3.5, *p* < .001). Parent age and household income did not correlate with critical thinking test scores.

College students with a liberal political affiliation (test mean score = 37.1) had significantly higher test scores relative to all other political affiliations (test mean score = 34.1) (t[143] = -2.2, *p* = .03). The other two affiliations—moderate and conversative—were not significantly related to critical thinking test results. College student age and household income did not significantly correlate with their critical thinking test scores.

## Discussion

This study assessed critical thinking about health of parents and college students in the US. Critical thinking about health includes the ability to evaluate claims about the effects and effectiveness of treatments, and has important public health implications, including empowering the public to make informed health choices. To the best of our knowledge, this was the first study of its kind in the U. S. Precisely, we measured critical thinking about health in the U. S. using the Claim Evaluation Tools item bank [23, 24]. We found that both parents and college students generally struggled to think critically about health. Additionally, we found that critical thinking about health was related to parent educational attainment and college student political affiliation. The current findings establish a baseline for critical thinking about health in the U. S., providing a foundation for future research and highlighting specific areas where the public faces the greatest challenges. Additionally, these results underscore the need for targeted interventions to enhance critical thinking skills in health-related contexts.

Parents’ overall critical thinking score (65%; 33.63 out of 52 correct responses) was slightly lower than college students pursuing a bachelor’s degree in the U.S (68%; 35.37 out of 52 correct responses). These scores are similar to those of Norwegian adults [18]. Of the three Key Concept categories for thinking critically about health, parents had the lowest scores in the category of “Making informed health choices.” Although this was not much different than their performance on critical thinking questions about understanding comparisons in healthcare research. Meanwhile, college students struggled most with applying Key Concepts in the category of understanding comparisons in research. Although scores in the category of “Making informed health choices” were not much better, either. In sum, both parents and college students struggled to think critically based on Key Concepts about choices in healthcare and understanding comparisons in healthcare research. Accordingly, future targeted interventions should prioritize these areas of critical thinking about health.

Fifty-five percent of parents in this study reported having a bachelor’s degree, (which is not uncommon for MTurk parent samples) [35], while the latest U. S. Census data indicates that only 23.5% of the public has a bachelor’s degree as their highest degree. One might assume that our sample might lead to results that over-estimated critical thinking relative to the general population of U. S. adults. However, when we examined the relationship between parent educational attainment and critical thinking, we found that having a bachelor’s degree was associated with worse critical thinking when compared to not having a bachelor’s degree. This finding merits further examination and replication as findings from studies in adjacent fields (e.g. health literacy, science literacy, and vaccine attitudes) appear to be mixed; some studies have found a similar relationship [38], the opposite relationship [28], or no relationship between educational attainment and variables similar to critical thinking about health (e.g., vaccine attitudes) [39–41]. Specifically, future studies could investigate potential mediating factors such as cognitive biases, overconfidence in knowledge, or trust in misinformation among individuals with higher educational attainment. Moreover, research should examine how contextual variables, such as exposure to quality health information or socio-cultural influences, interact with education to shape critical thinking about health. Expanding studies to include diverse populations with varied educational backgrounds will also provide more generalizable data.

Political affiliation is another sociodemographic factor that may impact critical thinking about health. We examined this potential relationship for college students only, since we did not ask parents about their political affiliation. We found that college students with a liberal political affiliation had significantly better critical thinking about health than those with conservative and moderate affiliations. Past studies in areas analogous to critical thinking about health have found similar relationships with political ideology [38, 42], as well as that cognitive sophistication or the ability to reason, sometimes supersedes the influence of political ideology [27, 42, 43]. This may suggest that sustained, targeted efforts to enhance the public’s critical thinking about health could empower individuals to make informed health choices, even admist diverse socio-cultural influences. Emphasizing skill development in reasoning may help bridge ideological divides and foster better health outcomes across populations.

### Implications for critical health literacy education and policy

Overall, this study’s main findings of low critical thinking about health in even among educated parents and college students point to a need for increased critical thinking supports in the U. S. This study demonstrated that the public may not possess the skills to understand research evidence at the same time that recent federal government efforts are increasing public access to this kind of information. To meet this need, two of the current authors developed and tested a brief critical thinking podcast intervention through a randomized controlled trial, which was found to be effective [44]. Future studies should further test targeted interventions for college students, perhaps ones embedded within their existing educational context. Researchers at Oslo Metropolitan University have been leading in this regard through their “Behind the Headlines” intervention for college students in Norway [45]. Notably, further developing the public’s critical thinking about health should serve alongside other approaches that tackle the systemic, root causes of health inequities; the onus should not and cannot solely rest on individuals. Relatedly, the latest *Healthy People 2030* (health.gov/healthypeople) by the U. S. Department of Health and Human Services, a 10-year plan to improve the health of people in the U.S, newly and distinctly emphasizes that organizations should also play a role in increasing the public’s health literacy [46]. Thus, public health strategies should integrate efforts to improve individual and organizational critical health literacy into broader systemic initiatives aimed at reducing structural barriers to equitable care. Policymakers can prioritize funding and implementation of community-based interventions that foster collaborative partnerships between healthcare organizations, educational institutions, and local governments.

## Strengths and limitations

One of the primary strengths of this study is that we examined a topic—critical thinking about health—with important public health implications for which we currently lack a strong research base. Furthermore, we examined critical thinking about health in two U. S. populations, parents and college students. Another strength of this study is that we used a validated measure of critical thinking about health.

This study also had limitations that bound the conclusions we can draw from the current assessment of critical thinking about health. Respondents with higher education were over-represented, and there is considerably low representation of racially/ethnically minoritized individuals. This is common with online-convenience samples; future studies should expand this work by including more representative samples of parents, especially parents from low-resource, community settings. Although past work supports the recruitment of parents on MTurk, especially in terms of data quality [35], there is a chance that these participants were completing multiple surveys, and that some of these were on related topics. Furthermore, there are limitations related to using a college student young adult sample. The study’s undergraduate college student sample represents the emerging/young adult population in the U. S. in general and important ways, as previously noted; however, they also deviate from the general young adult population. College students in the study were enrolled in a psychology course at a private university, and over 60% were freshmen from affluent and politically liberal/moderate backgrounds. As such, data from the present study serve as a first step in characterizing critical thinking about health in the U. S., and a reference point for future work seeking to expand evaluation to other demographic groups.

## Conclusion

This investigation provides empirical evidence for a need to increase critical thinking about health among U. S. parents and college students. U. S. parents and college students do not understand and apply many evidence-based practice principles that are crucial for engaging in critical thinking about health and empowering them to make informed health choices.

## Data Availability

The datasets used and/or analysed during the current study available from the corresponding author on reasonable request.

## Abbreviations

IHC: Informed Health Choices
MTurk: Mechanical Turk
